# Retraction Note: Sulfur availability regulates plant growth via glucose-TOR signaling

**DOI:** 10.1038/s41467-023-38298-8

**Published:** 2023-05-04

**Authors:** Yihan Dong, Marleen Silbermann, Anna Speiser, Ilaria Forieri, Eric Linster, Gernot Poschet, Arman Allboje Samami, Mutsumi Wanatabe, Carsten Sticht, Aurelio A. Teleman, Jean-Marc Deragon, Kazuki Saito, Rüdiger Hell, Markus Wirtz

**Affiliations:** 1 Centre for Organismal Studies (COS), University of Heidelberg, Im Neuenheimer Feld 360, 69120 Heidelberg, Germany; 2 Graduate School of Pharmaceutical Sciences, Chiba University, Chiba, 260-8675 Japan; 3 Center for Medical Research, University of Mannheim, 68167 Mannheim, Germany; 4 German Cancer Research Center (DKFZ), 69120 Heidelberg, Germany; 5 Laboratory of Genomes and Plant Development, Centre National de la Recherche Scientifique, University of Perpignan, 66100 Perpignan, France

Retraction to: *Nature Communications* 10.1038/s41467-017-01224-w, published online 27 October 2017

The authors are retracting this Article after they became aware that Fig. 2g, 3a, 3d, 4b-d, 5a, 5d, and Supplementary Fig. 1a were prepared in an inappropriate manner.

The blot images shown in Fig. 2g, 3a, 3d, 4b, 5a and 5d assess TOR activity and autophagy, and were produced by assembling different sections of the original blot images in a way that did not indicate the splicing and merging of lanes, and in some cases the loading controls shown did not correspond to the correct gels/blots. In addition, background sections of the plant images shown in Fig. 4c-d had been duplicated. Finally, the gel images shown in Supplementary Fig. 1a to confirm plant genotypes contain internal duplications and the original data cannot be found. Subsequent genotyping data has confirmed the genotype of the plants and the authors continue to believe that the data on TOR activity and autophagy are supported. The authors maintain full confidence in the metabolic profiling and transcriptomic data in the paper. Nevertheless, they recognize that the irregularities in image presentation could collectively decrease confidence in the conclusions.

The authors apologise to the scientific community for any confusion caused by these errors. All authors agree to this retraction.

